# Re-evaluation of the role of thoracoscopic esophagectomy as a Japanese-style radical surgery

**DOI:** 10.1007/s10388-016-0567-z

**Published:** 2017-01-03

**Authors:** Harushi Udagawa, Masaki Ueno, Shusuke Haruta, Tsuyoshi Tanaka, Aya Mizuno, Yu Ohkura

**Affiliations:** 1 Department of Gastroenterological Surgery, Toranomon Hospital, Okinaka Memorial Institute for Medical Research, 2-2-2, Toranomon, Minato-ku, Tokyo 105-8470 Japan; 2 Department of Gastroenterological Surgery, Toranomon Hospital, Minato-ku, Tokyo, Japan

**Keywords:** Esophageal cancer, Thoracoscopic esophagectomy, Video-assisted esophagectomy, Three-field lymphadenectomy, Minimally invasive surgery, Squamous cell carcinoma

## Abstract

**Purpose:**

To investigate the value of thoracoscopic surgery in radical esophagectomy with three-field lymphadenectomy.

**Materials and method:**

The subjects were 329 consecutive patients who, without preoperative chemoradiotherapy, underwent R0 radical esophagectomy with three-field lymphadenectomy for thoracic squamous cell esophageal cancers during 1998–2013. Open thoracotomy was applied in 212 (O), and thoracoscopic surgery in 117 (V). Survivals according to TNM Stages and Efficacy index (EI) were analyzed.

**Results:**

Hospital death rates of O/V were 1.9/0%. The survivals of V according to TNM Stages had significantly better prognosis in TNM6th cStage III and showed not worse prognosis in general. In the analysis using Cox proportional hazards model, “V or O” was a significant prognostic factor indicating better prognosis of V. More bilateral paratracheal lymph nodes along the recurrent laryngeal nerves tended to be classified as mediastinal instead of cervical in V. Efficacy index of mediastinal paratracheal nodes was higher in V than in O, while cervical lymphadenectomy maintained high EI.

**Discussion and conclusion:**

Though our series have limitations of retrospective study and substantial bias, the feasibility and safety of thoracoscopic esophagectomy with three-field lymphadenectomy was shown. Higher paratracheal lymph nodes along the recurrent laryngeal nerves could be dissected from the mediastinal side in V group. Thoracoscopic esophagectomy, which is regarded as minimally invasive surgery in other countries, is being accepted in Japan mainly in the expectation of more thorough and meticulous lymphadenectomy. At the same time, the dissection range is continuously re-evaluated for safer surgery maintaining radicality.

## Introduction

 In basic Japanese concept of multimodal treatment of esophageal cancer, radical surgery plays the main role in loco-regional control, and wide range of thorough lymphadenectomy is believed to be mandatory. The three-field lymphadenectomy is accepted as the standard procedure for this purpose [1]. On the other hand, thoracoscopic esophagectomy is rapidly prevailing in Japan. About 30% of esophagectomy in Japan is performed as thoracoscopic surgery [2, 3]. Thoracoscopic esophagectomy is generally regarded and accepted as a minimally invasive surgery in western countries [4, 5]. However, most of thoracoscopic surgeons in Japan try to perform thoracoscopic esophagectomy as radical as their own open surgery and think that thoracoscopic esophagectomy is even advantageous in accomplishing meticulous radical surgery [6, 7]. The purpose of this study was to clarify whether such high radicality was maintained and whether the value of three-field lymphadenectomy had been changed with the introduction of thoracoscopic surgery.

## Materials and method

A total of 419 consecutive patients who underwent curative radical esophagectomy with three-field lymphadenectomy for thoracic squamous cell esophageal cancers during 1998–2013 were first extracted for this study. Ninety patients who underwent neoadjuvant chemoradiotherapy (NACRT) were excluded because NACRT, which was applied to patients with bulky T3 or suspicious T4 tumors including metastatic lymph nodes, seemed to be the largest bias between the groups of patients with open surgery (NACRT: 85) and thoracoscopic surgery (NACRT: 5). Consequently, subjects of the study were 329 patients, 201 in open thoracotomy group (O group), and 117 in video-assisted (=thoracoscopic) surgery group (V group). The open esophagectomy with three-field lymphadenectomy was performed as had been described by Akiyama [8]. The video-assisted esophagectomy was done with essentially similar technique to what had been described by Osugi [9]. The video-assisted esophagectomy was started in September of 2006. Survivals according to TNM Stages and Efficacy index [10] (EI: metastatic rate % × 5-y survival% × 1/100) were analyzed. The characteristics of the 329 subjects are listed in Table 1. The sixth edition of TNM classification [11] was applied in clinical TNM evaluation because number of clinically positive lymph nodes in preoperative staging was not available in patients before 2009. Pathological TNM staging was done according to the seventh edition of TNM [12]. The analysis on individual lymph node stations was done according to the latest classification by Japan Esophageal Society [13].

**Table 1:** Characteristics of the subjects

Subjects	329 patients		
Interval	1998–2013		
Diagnosis	Thoracic esophageal squamous cell carcinoma		
Neoadjuvant Tx.	None or chemotherapy (CRT excluded)		
Operation	Three-field lymphadenectomy, R0 resection		
Group	O (open)	V (video-assisted)	*P* value
Number	212	117	
Age	62.3 ± 8.5	62.4 ± 8.3	.937**
Sex			
Male/female	190/22	103/14	.713
Location			
U/M/L	29/127/56	23/66/28	.360*
Preoperative treatment			
None/Chemo	166/46	49/68	<.001
cT (TNM6th)			
1a/1b/2/3/4	3/68/42/95/4	5/38/39/34/1	.009*
cN (TNM6th)			
0/1	62/150	43/74	.175
cM (TNM6th)			
0/1a/1b	175/8/29	107/2/8	.087*
cSt (TNM6th)			
I/IIA/IIB/III/IVA/IVB	39/23/42/71/8/29	27/16/40/24/2/8	.006*
pT (TNM7th)			
0/1a/1b/2/3/4a/4b	1/15/71/20/100/5/0	0/15/46/25/31/0/0	.001*
pN (TNM7th)			
0/1/2/3	79/62/43/28	41/46/24/6	.066*
pM (TNM7th)			
0/1	173/39	102/15	.216
pSt (TNM7th)			
IA/IB/IIA/IIB/IIIA/IIIB/IIIC/IV	41/10/26/30/31/22/13/39	19/9/10/30/25/7/2/15	.023*
Hospital death	4 (1.9%)	0	.301

In pT(TNM7th) column, ypT0 is listed as T0

In pSt(TNM7th) column, ypT0N0M0 is included in IA, and ypT0N1M0 is included in IB

Statistical significance * Pearson’s Chi-square test, ** *t* test, *others* Fisher’s exact test

Pearson’s Chi-square test, Fisher’s exact test, and *t* test were used for distributional and numerical comparison. The survival curves were estimated according to the Kaplan–Meier method, and overall survival distributions were compared by use of the log-rank test. Cox proportional hazards model, forward stepwise method by likelihood ratio with the condition of .05 for addition and .1 for deletion, was applied to investigate prognostic factors. IBM SPSS Statistics 23^®^ was used for data analysis.

## Results

Hospital death rates of O/V were 1.9/0% (Table 1). Survivals according to cTNM6th Stages at first presentation of the patients were not statistically different between the two groups except among cSt III patients where V group showed better survival (Table 2, upper). The backgrounds of the 2 groups in this stage are not the same. Preoperative treatment was significantly different (None/Chemo of O was 55/16, while that of V was 5/19.), though preoperative treatment was not a significant prognostic factor in univariate analysis (Table 3). There was no significantly different distribution in other factors (data not shown). V group tended to show better survival in almost every pTNM7th stage though without statistical significance (Table 2, middle). Table 2 shows that V group had better survival than O group in TNM7th pN0 patients, the similar tendency in pN1, but did not in pN2 and pN3.

**Table 2:** Five-year survival of O group and V group according to TNM factors

	O (%)	V (%)	*P* (Log rank)
	5y.s. (S.E.)	5y.s. (S.E.)	
TNM6th cStage			
I	86.9 (5.5)	89.3 (7.5)	.594
IIA	65.2 (9.9)	78.7 (11.0)	.409
IIB	78.6 (6.3)	83.5 (6.9)	.370
III	50.9 (6.0)	73.0 (10.5)	.026
IVA	25.0 (15.3)	– (–)	.685
IVB	64.4 (9.0)	70.0 (18.2)	.836
TNM7th pStage			
IA	92.9 (4.1)	100 (–)	.239
IB	80.0 (12.6)	100 (–)	.194
IIA	72.7 (8.8)	100 (–)	.081
IIB	73.3 (8.1)	86.1 (7.7)	.266
IIIA	67.0 (8.6)	61.8 (10.8)	.701
IIIB	57.1 (10.8)	75.0 (21.7)	.290
IIIC	27.7 (13.5)	0 (–)	.300
IV	35.9 (7.7)	70.0 (12.8)	.125
TNM7th pN			
0	84.8 (4.1)	100 (–)	.011
1	64.0 (6.1)	74.2 (8.0)	.083
2	59.5 (7.6)	73.5 (9.4)	.179
3	23.2 (8.2)	25.0 (20.4)	.796

**Table 3:** Results of univariate Cox regression model

Covariate	B	SE	*P*	HR
V/O	**−.837**	**.261**	**.001**	**.433**
Period (L/F)	−.336	.213	.116	.715
PreTx.				
CT/NO	.001	.208	.995	1.001
Location			.339	
U/M	.161	.268	.548	1.175
L/M	.307	.210	.145	1.359
**Age (+1)**	**.032**	**.012**	**.008**	**1.032**
Sex(M/F)	.424	.347	.222	1.528
**cT (TNM6th)**			**.000**	
**T1a/T2**	**−1.141**	**1.020**	**.263**	**.320**
**T1b/T2**	**−.480**	**.279**	**.085**	**.619**
**T3/T2**	**.456**	**.235**	**.052**	**1.577**
**T4/T2**	**1.487**	**.614**	**.015**	**4.424**
**cN (TNM6th)**				
**N1/N0**	**.749**	**.226**	**.001**	**2.114**
**cM (TNM6th)**			**.004**	
**M1a/M0**	**1.207**	**.393**	**.002**	**3.343**
**M1b/M0**	**.417**	**.265**	**.116**	**1.517**
**pT (TNM7th)** ^*1^			**.000**	
**T1a/T2**	**−.500**	**.548**	**.362**	**.607**
**T1b/T2**	**−.119**	**.367**	**.745**	**.887**
**T3/T2**	**.841**	**.340**	**.013**	**2.320**
**T4a/T2**	**1.970**	**.550**	**.000**	**7.171**
**pN (TNM7th)**			**.000**	
**N1/N0**	**.918**	**.274**	**.001**	**2.504**
**N2/N0**	**1.188**	**.289**	**.000**	**3.279**
**N3/N0**	**2.199**	**.301**	**.000**	**9.019**
**pM (TNM7th)**				
**M1/M0**	**1.021**	**.210**	**.000**	**2.777**

*B* regression coefficient, *SE* standard error, *HR* hazard ratio = Exp(B)

*Period F* operated before we introduced video-assisted esophagectomy

*Period L* operated after we introduced video-assisted esophagectomy

The bold values indicate that the factors are statistically significant in univariate analyses and will be included in the next multivariate analyses

* 1 One pT0 patient in O group after chemotherapy is excluded to maintain appropriate statistical analysis

Twelve clinicopathological factors were examined as univariates for Cox proportional hazard model and found to be statistically significant except preoperative treatment, period of operation, tumor location, and sex (Table 3). Therefore, we decided that 8 factors with statistical significances should be included in the multivariate analysis. Because cTNM6th factors and pTNM7th factors should have strong correlation, Cox proportional hazards model was applied to the data set of 5 patient characteristics including cTNM6th factors but excluding pTNM7th factors. This pointed out that V/O status, cT, cM, and age were significant prognostic factors (Table 4, upper). When the same analysis was done to the data set of 5 patient characteristics including pTNM7th information but excluding cTNM6th pointed out that V/O status, pT, pN, and age were significant prognostic factors (Table 4, lower).

**Table 4:** Covariates in the equation of the Cox regression model

Covariate	B	SE	P	HR
Study with cTNM6th factors				
V/O	−.788	.265	.003	.455
cT6th			.000	
T1a/T2	−.904	1.021	.376	.405
T1b/T2	−.577	.281	.041	.562
T3/T2	.334	.239	.164	1.396
T4/T2	1.445	.618	.019	4.240
cM6th			.46	
M1a/M0	.986	.400	.013	2.690
M1b/M0	.050	.273	.859	1.052
Age (+1)	.036	.012	.003	1.036
Study with pTNM7th factors^*1^				
V/O	−.629	.269	.019	.533
pT7th			.011	
T1a/T2	−.066	.562	.907	.936
T1b/T2	−.162	.370	.662	.850
T3/T2	.578	.345	.094	1.782
T4a/T2	.939	.579	.105	2.557
pN7th			.000	
N1/N0	.920	.281	.001	2.510
N2/N0	1.062	.303	.000	2.891
N3/N0	2.143	.334	.000	8.526
Age (+1)	.052	.012	.000	1.053

*B* regression coefficient, *SE* standard error, *HR* hazard ratio = Exp(B)

* 1: One pT0 patient in O group after chemotherapy is excluded to maintain appropriate statistical analysis

Number of dissected mediastinal nodes was significantly less in V group (O/V: 42.4/36.0) (Table 5, upper). Evident decrease in number of dissected lymph nodes in the station 106pre, which had the least EI value of .30, was observed (Table 5, lower). The number of bilateral mediastinal paratracheal nodes dissected was a little more in V group, though not statistically significant, and the number of bilateral cervical paratracheal nodes was significantly less in V (Table 5, lower). The percentage of paratracheal recurrent nerve chain nodes dissected from the mediastinal side in both groups is shown in Table 6. The percentage was higher on both sides in V group.

**Table 5:** Average numbers of dissected lymph nodes in O group and V group

Field	O	V	*P* (*t* test)
Cervical	45.2 ± 16.2	42.9 ± 14.9	.189
Mediastinal	42.4 ± 13.4	36.0 ± 12.3	<.001.
Abdominal	29.1 ± 11.6	29.2 ± 11.6	.973
3 fields	116.6 ± 28.8	108.0 ± 28.4	.010
Station	O	V	P
106pre	6.1 ± 5.1	2.4 ± 2.8	<.001.
101(R + L)	6.3 ± 4.0	5.3 ± 4.1	.033
106rec(R + L)	9.9 ± 4.9	10.9 ± 5.6	.098

**Table 6:** Percentage of paratracheal lymph nodes dissected from the mediastinal side in O group and V group

Side	O (%)	V (%)	*P* (*t* test)
Right	58.5 ± 26.5	66.4 ± 25.1	.009
Left	63.8 ± 23.8	69.9 ± 23.1	.024
Bilateral	61.2 ± 19.8	67.8 ± 17.4	.003

Percentage of paratracheal lymph nodes dissected from the mediastinal side = number of 106rec nodes/(number of 106rec nodes + number of 101 nodes)

EI value of mediastinal paratracheal node station (106rec) was higher in V than in O. This was more evident on the left side (Table 7). The EI’s in both O and V groups for cervical, mediastinal, and abdominal lymphadenectomy were all very high, and no decrease in EI of cervical lymphadenectomy from O group to V group was observed (Table 8).

**Table 7:** EI’s of paratracheal (recurrent nerve chain) lymph node stations in O group and V group

Station	O or V	Met. r (%)		5-y s (%)	Efficacy index
101R	O	10.4	*P* = .031*	45.0	4.7
	V	3.4		66.7	2.3
106recR	O	22.2		51.1	11.3
	V	22.2		62.2	13.8
101L	O	6.6		50.0	3.3
	V	5.1		83.3	4.2
106recL	O	10.4	*P* = .005*	52.5	5.5
	V	22.2		71.5	15.8

* Fisher’s exact test

**Table 8:** EI’s of the three fields in O group and V group

Field	O or V	Met. r (%)		5-y s (%)	Efficacy index
Cervical	O	17.1		67.3	11.5
	V	25.5		46.2	11.8
Mediastinal	O	47.6		51.5	24.5
	V	52.1		67.5	35.2
Abdominal	O	38.2	*P* = .021*	46.6	17.8
	V	25.6		67.4	17.3

* Fisher’s exact test

## Discussion

Thoracoscopic esophagectomy, which is regarded as minimally invasive surgery in other countries, is being accepted in Japan very rapidly. According to the NCD report, about 30% of all esophagectomies performed in Japan in 2011 were done as video-assisted surgery. However, most Japanese surgeons performing video-assisted esophagectomy have the impression that the largest advantage of video-assisted esophagectomy is not its less invasiveness but the expectation of more thorough and meticulous lymphadenectomy. In our series, most of the analyses on prognosis showed tendency of improvement with video-assisted surgery. Taking the large bias of this study design into account, we feel at least we can conclude that the introduction of video-assisted surgery has not worsened the prognosis of our radical esophagectomy.

Bilateral paratracheal lymph nodes, which are also known as recurrent nerve chain nodes, are most important nodes to be dissected in radical esophagectomy for thoracic esophageal cancer. They are uninterruptedly located from the mediastinum to the cervical area. Our data showed that more paratracheal lymph nodes tend to be dissected from mediastinal side and fewer nodes were dissected from the cervical side in thoracoscopic esophagectomy. Consequently, EI’s of mediastinal paratracheal lymph node stations are higher in V group than in O group. The large increase in EI of the station 106recL from O to V might show not only the fact that more 106recL nodes including nodes in more cervical location can be dissected but also that more complete lymph node dissection of this station can be achieved in thoracoscopic surgery.

Although more nodes in cervical paratracheal station (101) can be dissected from the mediastinal side as 106rec in thoracoscopic esophagectomy, high EI value of cervical lymph node dissection is still maintained because of better 5-year survival of cervical node-positive patients in V group. It might mean that the combination of video-assisted mediastinal dissection + cervical lymphadenectomy be superior to the combination of conventional open mediastinal dissection + cervical lymphadenectomy in terms of completeness. At least we can say that three-field lymphadenectomy remains as the standard operative procedure even in the era of video-assisted surgery and multimodal treatment.

Because this study is a retrospective study, it should contain biases. The largest limitations are that V group was operated later than 2005 and that the distribution of clinical and pathological factors of the patients in both groups was different. The latter can be adjusted to some extent by stratifying patients according to these clinical and pathological factors. However, the former bias cannot be adjusted. We are looking forward to the coming results of JCOG1409, the phase III study among thoracoscopic esophagectomy and open esophagectomy.

Neoadjuvant chemoradiotherapy followed by surgery for resectable advanced thoracic esophageal cancer is widely accepted in all over the world. On the other hand, preoperative chemotherapy followed by surgery is regarded as standard in Japan. This strategy has yielded very good prognostic result as shown here. In this study, patients with NACRT were excluded because it was strongly associated with advanced T-stage, poorer prognosis, and open surgery. There is no direct evidence to compare NACRT and neoadjuvant chemotherapy particularly when the surgery includes Japanese-style extended lymphadenectomy. Japan Esophageal Oncology Group is executing a three-armed randomized controlled trial, JCOG1109 [14], to give answer to this question.

## Conclusion

 Japanese-style three-field lymphadenectomy pursuing best loco-regional control by surgery is feasibly and safely performed as thoracoscopic surgery. Thoracoscopic esophagectomy, which is regarded as minimally invasive surgery in other countries, has ability to realize more thorough and radical lymphadenectomy. This phenomenon was observed in the area along the recurrent laryngeal nerves. This increased radicality seems to improve long-term survival of the patients operated thoracoscopically. However, clear evidence of superiority of thoracoscopic surgery to conventional open surgery cannot be obtained until a phase III trial on this issue such as JCOG 1409 becomes available.
